# Undiagnosed HIV, hepatitis B, and hepatitis C infections in people with severe psychiatric disorders in Ethiopia

**DOI:** 10.1186/s12879-020-4907-1

**Published:** 2020-02-27

**Authors:** Getinet Ayano, Kibrom Haile, Abel Tesfaye, Kelemua Haile, Sileshi Demelash, Mikias Tulu, Belachew Tsegaye, Melat Solomon, Alem Kebede, Aynalem Biru, Habte Birhanu, Gebresilassie Zenawi, Yodit Habtamu, Esias Kibron, Seneshet Eshetu, Meseret Sefiw, Dawit Assefa, Zegeye Yohannes

**Affiliations:** 1 Research and Training Department, Amanuel Mental Specialized Hospital, PO Box 171, Addis Ababa, Ethiopia; 2 School of Public Health, Curtin University, Perth, Westen Australia, Australia; 3 Department of medicine, Hawassa University, Hawassa, Ethiopia; 4 Ethiopian public health institute, Addis Ababa, Ethiopia

**Keywords:** Severe psychiatric disorder, Undiagnosed, HIV infection, HBV infection, And HCV infection

## Abstract

**Background:**

Worldwide, there is limited epidemiologic evidence on the seroprevalence of undiagnosed chronic viral infections including HIV, hepatitis B virus (HBV) and hepatitis C virus (HCV) infections among patients with severe psychiatric disorders. To our knowledge, this is the first study to explore and compare undiagnosed seroprevalence rates of HIV, HBV, and HCV infections among patients with severe psychiatric disorders.

**Method:**

In this study, we included a random sample of 309 patients with severe psychiatric disorders selected by systematic sampling technique. We used a structured clinical interview for DSM-IV (SCID) to confirm the diagnosis of severe psychiatric disorders among the participants. Binary and multivariable logistic regression models, adjusting for the potential confounding factors was used to explore the potential determinants of chronic viral infections.

**Result:**

The prevalence estimates of HIV infection among patients with severe psychiatric disorders in this study (3.24%) was roughly 3 times the estimated population prevalence of HIV infection in Ethiopia (1.1%). This study showed that the prevalence rates of HBV and HCV infections among patients with severe psychiatric disorders were 4.85 and 1.29%, respectively. Our results also showed that among patients with chronic viral infections, HIV, HBV and HCV, 76.92, 60, 80, and 75% respectively were undiagnosed. Regarding associated factors, the presence of chronic viral infection was found to be significantly associated with the age of the participants (ranging between 30 and 40 years) after adjusting for the possible confounding factors [AOR = 3.95 (95%CI.18–13.17**)**].

**Conclusion:**

Even though the prevalence estimates of HIV (3.24%), HBV (4.85%), and HCV (1.29%) infections were high among patients with severe psychiatric disorders, the majority of them remained undiagnosed. HBV was found to be the commonly undiagnosed infection (4 out of 5) followed by HCV (3 out of 4) and HIV (6 out of 10). The present study provided evidence of a significant association between the age of the participant (between 30 and 40 years) and chronic viral infections in patients with severe psychiatric disorders. Increasing the awareness of psychiatry professionals and early screening, as well as interventions of chronic viral infections among patients with severe psychiatric disorders are imperative.

## Background

Severe psychiatric disorders such as schizophrenia, bipolar, schizoaffective, and depressive disorders are important contributors to the global burden of disease and they are among the common leading causes of the global burden of morbidity and early deaths [1, 2]. Severe mental disorders including schizophrenia and psychosis affect 1 to 2% of the general adult population [3]. The lifetime prevalence of Bipolar I disorders in Ethiopia was estimated to be 0.6% in males and 0.3% in females [4]. The reported lifetime prevalence estimate of schizophrenia in Ethiopia was 4.7/1000 [5] and it was 1.2% for a major depressive disorder [6].

Epidemiologic evidence shows that a considerable percentage of people with severe psychiatric disorders had comorbid viral infections such as human immunodeficiency virus infection (HIV) 6.2–29.10% [7–10], hepatitis B virus (HBV) 7.45–47.5% [10–13], as well as hepatitis C virus (HCV) 6.2–29.8% [7, 10, 13, 14] infections at some point in their lives. These prevalence estimates are considerably higher than the reported prevalence in the general population [15–18]. In Ethiopia, the estimated prevalence of HIV, HBV, and HCV were 0.66% [15], 6.3% [16], and 3% [16] respectively.

Scientific studies also showed that a large number of patients with HIV, HBV, as well as HCV infections in the general population remained undiagnosed [19, 20]. For example, in a study performed in Europe, the estimated magnitude of undiagnosed HIV in the general population was 30% [19]. In another study conducted in the USA, about 40 to 50% of cases of HCV remain undiagnosed [21]. Regarding undiagnosed rates of HBV, the reported estimate of the prevalence of undiagnosed cases of HBV infection in the general population in a study conducted England was found to be 16% [22].

Even though there are not enough supportive data on the prevalence estimate of undiagnosed viral infections among people with severe psychiatric disorders, it is assumed that the prevalence rates of undiagnosed HIV, HBV, and HCV believed to be considerably higher in patients with severe psychiatric disorders as compared to general population rates due to the nature of the illness which impairs their decision-making capacity [23, 24], makes them to involve in risky sexual behaviors [25, 26], and the reported higher magnitude of undiagnosed medical conditions (up to 80%) in people with mental illness [27, 28].

In the context of the above issues, this study aimed to estimate the prevalence of undiagnosed chronic viral infections including HIV, HBV, and HCV infections among patients with severe psychiatric disorders. Since this is the first study that aimed to explore the prevalence estimates of undiagnosed HIV, HBV, and HCV infections among patients with severe psychiatric disorders, the findings of the study will help to better understand the prevalence rates of undiagnosed HIV, HBV, and HCV infections among patients with severe psychiatric disorders and to provide possible recommendations based on the findings.

## Methods

### Study design

A cross-sectional seroepidemiological survey was conducted among patients with severe psychiatric disorders recruited from the outpatient clinic at Amanuel Mental Specialized Hospital, Addis Ababa, Ethiopia from May 1, 2017, to July 30, 2017.

### Eligibility criteria

To be included in this study the participant had to satisfy the following criteria: first, being an adult (aged over 18 years); second, had a diagnosis of one of the severe psychiatric disorders such as schizophrenia, bipolar, schizoaffective, and depressive disorders by the structured clinical interview for DSM- ΙѴ-TR axis Ι disorders (SCID) criteria. Additionally, for those randomly selected participants, the data collectors assessed the capacity of a person to consent to participate in the study. If the person had a capacity and given informed consent, they were considered eligible and further assessment was administered by the data collectors.

### Sampling procedure

The present study is the part of comorbidity study in a specialized psychiatric setting in Ethiopia. Thus, the sample size was calculated using Epi-info version 7 with a 95% CI, 5% margin of error and taking the prevalence of the comorbid medical disease in people with severe psychiatric disorders nearly 80% [28, 29] and based on this the estimated sample size was 246. Considering a 30% non-response rate a total sample of 320 patients with severe psychiatric disorders were included.

A systematic random sampling technique was employed to select the study participants. We identified the sampling interval by dividing the total patients with severe psychiatric disorders who had the treatment and follow up during the data collection period by total sample size which was 11. We used a lottery method to select the first participant, and the remaining participants were selected at a regular interval as suggested by the systematic sampling method.

**Fig. 1:**
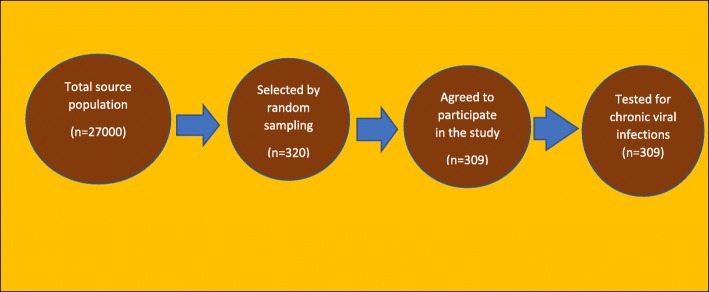
Schematic representation of sample size and sampling procedure

Those participants who have been randomly selected to be part of the survey but who refused to take part in the study were considered non-respondents. The non-response rate in the current study was found to be 3.44%. (See Fig. 1).

### Measures

#### Screening for severe psychiatric disorders

In this study, severe psychiatric disorders were assessed by using the structured clinical interview for DSM- ΙѴ-TR axis Ι disorders (SCID) [30]. SCID which is a validated and standard diagnostic tool to assess DSM-IV-axis I disorders (major psychiatric disorders) and it has been extensively utilized to assess psychiatric disorders in previous studies in Ethiopia [31–35].

#### Screening for HIV, HBV, and HCV infections

The previous diagnosis of HIV, HBV, as well as HCV infections, were taken from the chart record of each participant. To do this we included a questionnaire assessing whether or not the participants had received a diagnosis of HIV, HBV, as well as HCV infections from their chart record. The screening of the current HIV, HBV, as well as HCV infections was conducted by trained laboratory professionals after thorough interview and taking the willingness of the patients to participate. Five milliliters of the blood sample was aseptically collected from each participant by medical laboratory professionals. The samples were then tested for antibody to Hepatitis C virus (anti-HCV) and Hepatitis B antigen (HBsAg) using a rapid immunoassay designed to test HBV markers (HBsAg) [36, 37] for the diagnosis of HCV and HBV infection, respectively. From each collected samples anti-HCV and HBsAg were tested by using an enzyme-linked immunological assay (ELSA) following manufacturers protocol [37, 38]. Similarly, we screened for HIV diagnosis by using a rapid HIV diagnostic test kits according to the manufacturer’s protocol [39, 40]. We also adhered the national guide for conducting an HIV test in Ethiopia [40]. The Ethiopian ministry of health currently recommends the serial rapid testing algorithm with determine, STAT-PAK, and Uni-gold for the diagnosis of HIV infection [40]. All serum samples were tested for HIV (1 + 2) antibody using the standard operating procedure (SOP) for HIV *1/2* STAT- PAK test in Ethiopia [40, 41]. All testing was conducted by qualified and experienced laboratory professionals.

The patients with no previous diagnosis but who received current positive tests for HIV, HBV, as well as HCV infections, were linked to the medical department which gives basic medical services including HIV testing, counseling, and treatment as well as care and treatment for the other medical conditions to handle the current diagnosis.

#### Sociodemographic and other factors

For this study, the sociodemographic and clinical characteristics such as age, sex, residence, marital status, educational status, ethnicity, religion, suicide, duration of the illness, history of relapse and hospitalizations were collected from each of the participants. All data was collected using trained assessors (masters level psychiatry professionals) assigned to evaluate the participants based on SCID criteria.

#### Data quality control

In the current study, to assure the quality of the data, the data collector (psychiatry professionals) who have adequate knowledge and experience about DSM- ΙѴ-TR were recruited. Additionally, training was delivered on how to complete the questionnaire, sampling procedure, inclusion criteria, ethical consideration, data collection procedure as well as the details of SCID for the data collectors and supervisors. The questionnaire was pretested before the actual data collection and the necessary modification was made. Two Supervisors followed the data collectors and the necessary correction was undertaken when needed. The collected data were reviewed and checked for completeness and relevance by the supervisor and principal investigator each day.

### Statistical analysis

Stata version 14 software package was used to conduct statistical analysis. Frequency/percentage was used to express categorical variables and mean (standards deviations) was used for continues variables. We carried out bivariate and multivariate logistic regression to look at the association between outcome and explanatory variables. OR with 95% CI was used to measure the strength of the association and a *P*-value less than 0.05 was considered as statistically significant.

### Ethical consideration

In accordance with the given roles and national research ethics guidelines [42], the human research and ethics committee (HREC) of Amanuel Mental Specialized Hospital (Research and training department) reviewed and approved the study protocol. Participants who had the capacity to consent were involved in the study. Every participant signed a written consent after a clear and detailed explanation of the purpose, objectives, significance, benefits, and harms of participation, as well as confidentiality of the collected information to each of the study participants. The survey teams (coordinators and investigators) made sure that the anonymity and confidentiality of the participant included in this serologic study were confirmed.

## Results

### Sociodemographic characteristics of the participants

Table 1 shows the sociodemographic characteristics of the participant included in this study. A total of 309 participants with severe psychiatric disorders including schizophrenia (*n*==135), schizoaffective (*n* = 28), bipolar (*n* = 54), and depressive (*n* = 92) disorders were involved in the current study, yielding a response rate 96.56%. The mean (SD) age and duration of the participants were 36.19 (10.45) and 10.04 (8.66) years, respectively. Nearly two-thirds of the participants 202 (65.37%) were males, 202 (65.37%) were single, 118 (38.19%) attended secondary school, nearly half 157 (51.47%) were Orthodox Christians, and the majority of the participants 235 (76.05%) were from urban areas.

**Table 1:** Sociodemographic characteristics of the participants with severe psychiatric disorders in Addis Ababa, Ethiopia (*n* = 309)

Characteristics	Frequency	Percentage
Age		
30 or less	110	35.60
30 to 40	106	34.30
41 and more	93	30.10
Sex		
Male	202	65.37
Female	107	34.63
Educational status		
Uneducated	30	9.71
Primary	103	33.33
Secondary	118	38.19
Higher	58	18.77
Religion		
Muslim	87	28.16
Orthodox	157	51.46
Protestant	57	18.54
Others	6	1.94
Ethnicity		
Amhara	95	30.74
Gurage	82	26.54
Oromo	91	29.45
Others	41	13.27
Marital status		
Single	202	65.37
Married	74	23.95
Divorcee/widowed	33	10.68
Residence		
Urban	235	76.05
Rural	74	29.95
SPD type		
Schizophrenia	135	43.69
Bipolar disorder	54	17.48
MDD	92	29.77
Schizoaffective disorders	28	9.06

### The prevalence of chronic viral infections in patients with severe psychiatric disorders

In the current study, the overall prevalence of chronic viral infections (includes the three blood born viruses ─ HIV, HBV, and HCV combined) was found to be 8.41% (95%CI 5.74–12.06).

Regarding the specific types of viral infections, the prevalence of HIV, HBV, and HCV among people with SPD were found to be 3.24% (95%CI 1.74–5.93), 4.85% (95%CI 2.94–7.92), and 1.29% (95%CI 0.48–3.42), respectively.

Moreover, in this study, the prevalence of any hepatitis virus (HBV and HCV combined) was found to be 5.83%. Also, we found similar prevalence estimates for the coinfection of HIV and HBV (0.32%), HBV and HCV (0.32%), as well as HIV and HCV (0.32%). The magnitude of the triple infection (HIV, HBV, and HCV on the same person) in this study was found to be 0.00% (Table 2).

**Table 2:** The prevalence of infectious diseases among patients with severe mental disorders in central Ethiopia, *n* = 309

Illness	Cases	Prevalence (%)	95%CI
Overall infectious disease	26	8.41	5.78–12.06
HIV	10	3.24	1.74–5.93
HBV	15	4.85	2.94–7.92
HCV	4	1.29	0.48–3.42
HBV or HCV	18	5.83	3.699.08
HIV and HBV coinfection	1	0.32	0.05–2.28
HIV and HCV coinfection	1	0.32	0.05–2.28
HBV and HCV coinfection	1	0.32	0.05–2.28
Triple infection	0	0.00	–

Key: *HIV* Human Immune Deficiency Virus, *HIB* Hepatitis B virus, *HVC* Hepatitis C virus

### The prevalence of undiagnosed chronic viral infections in patients with severe psychiatric disorders

In this study, the prevalence of the undiagnosed chronic viral infections among patients with severe psychiatric disorders was 6.47% from the total participants and among patients with chronic viral infections, 76.92% were undiagnosed.

We found that four participants had undiagnosed-cases of HIV (1.94% of the total patients with severe psychiatric disorders and 60% among HIV cases).

We also found that four participants had undiagnosed-cases of HBV (3.88%) of the total patients with severe psychiatric disorders and 80% among HBV cases).

**Fig. 2:**
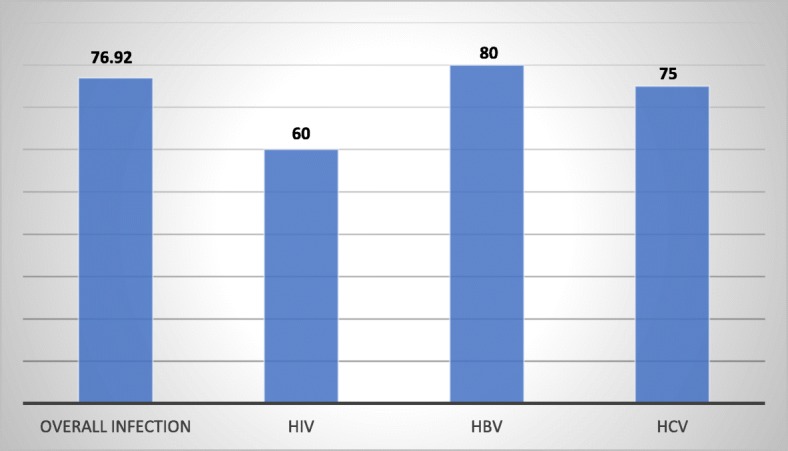
The rates of misdiagnosis of infectious diseases (in percenatge)

Furthermore, four participants had undiagnosed-cases of HCV (0.97%) among the total patients with severe psychiatric disorders and 75% among HCV cases) (Table 3 and Fig. 2).

**Table 3:** The prevalence of undiagnosed infectious diseases among patients with severe mental disorders in central Ethiopia, *n* = 309

Illness	Chart diagnosis, n (%)	Real diagnosis, n (%)	Undiagnosed illness from the total, n (%)	Undiagnosed illness from the cases, n (%)
Overall infectious disease	7 (2.77%)	26 (8.41)	20 (6.47)	20 (76.92)
HIV	4 (1.29)	10 (3.24)	6 (1.94%)	6 (60.00)
HBV	3 (0.97)	15 (4.85)	12 (3.88)	12 (80.00)
HCV	1 (0.32)	4 (1.29)	3 (0.97)	3 (75.00)

Key: *HIV* Human Immune Deficiency Virus, *HIB* Hepatitis B virus, *HVC* Hepatitis C virus

### Factors associated with undiagnosed chronic viral infections in patients with severe psychiatric disorders

In this study, after adjusting the model for the possible confounding factors, the age of the participant was found to be a significant predictor of chronic viral infections. The odds of having chronic viral infections were increased by 3.95 [AOD = 3.95 (95%CI.18–13.17**)**] for those participants in the age range between 30 and 40 years as compared with the age group between 18 and 30 years (Table 4).

**Table 4:** Factors associated with infectious disease in people with severe mental disorders, Addis Ababa, Ethiopia

Characteristics	Misdiagnosis		Crude odds ratio (95%CI)	Adjusted odds ratio (95%CI)
	Yes	No		
Gender				
Female	13	94	2.01 (0.89–4.50)	1.98 (0.83–4.76)
Male	13	189	1	1
Age				
18–30	4	106	1	1
31–40	14	92	4.03 (1.28–12,68)*	3.95 (1.18–13.17)*
≥ 40	8	85	2.49 (0.72–8.56)	1.17 (0.58–2.36)
Residence				
Rural	3	71	1	1
Urban	23	212	2.56 (0.75–8.80)	0.38(0.10–1.45)
Marital status				
Single	12	190	1	1
Married	8	66	1.91 (0.75–4.90)	2.39 (0.86–6.49)
Divorce/widowed	6	27	3.51 (1.22–10.15)*	2.58(0.80–8.33)
Misdiagnosed severe psychiatric disorder				
Correct diagnosis	14	174	–	**–**
Misdiagnosis	12	109	1.37 (0.61–3.06)	1.79 (0.74–2.07)
Relapse				
Relapsed	19	207	1.00 (0.40–2.45)	0.77 (0.28–2.11)
No relapse	7	76	1	
Admission				
Admission	15	181	0.77(0.34–1.74)	0.83 (0.33–2.06)
No admission	11	102	1	1
WHODAS score			0.87(0.38–1.97)	0.86 (0.36–2.03)

* Significant association (*p*-value < 0.05)

## Discussion

### Main findings

To the best of our knowledge, this is the first study that examined the epidemiology of undiagnosed chronic viral infections including HIV, HBV, and HCV among patients with severe psychiatric disorders. The main purpose of the study was to estimate the prevalence of undiagnosed chronic viral infections in patients with severe psychiatric disorders in a specialized psychiatric setting. The results of this study revealed that a remarkable proportion of people with severe psychiatric disorders had undiagnosed chronic viral infections and the estimated prevalence of undiagnosed HIV, HBV and HCV were considerably higher than the reported prevalence estimates in the general population. Among patients with chronic viral infections, HIV, HBV and HCV, 76.92, 60, 80, and 75% respectively were undiagnosed. These findings suggest the urgent need to improve the awareness of psychiatry professionals as well as early screening and management of chronic viral infections among patients with severe psychiatric disorders.

### The prevalence of chronic viral infections in people with severe psychiatric disorders

In this study, the prevalence of chronic viral infections such as HIV, HBV, and HCV was considerably lower than the reported global magnitude of those viral diseases among patients with severe psychiatric disorders from previously published studies [10, 43, 44]. For example, a recent meta-analysis showed that the prevalence of HIV, HBV, and HCV among people with severe psychiatric disorders was 7.59, 15.3, and 7.21% [10], respectively which is significantly higher than the reported prevalence of HIV (3.2%), HBV (4.85%) as well as HCV (1.29%) in this study. As compared with the findings of other countries the prevalence of HIV was in this study was consistent with the magnitude of HIV among people with severe mental illness in central and South America (2.74%) [43] but lower than the reported magnitude of HIV in Africa (19.2%) [43] and North America (6.0%) [43]. In contrast, our findings are lower than the meta-analysis reports of the magnitude of HIV among people with severe mental illness in Europe (1.9%) [43] and Asia (1.5%) [43]. However, the prevalence of HBV and HCV in this study were consistent with the reported magnitude of HBV and HCV from the developing countries but considerably lower than the magnitude of HBV and HCV from the developed countries [10, 43]. The possible reasons for the lower magnitude of HBV, HBV and HCV from the reported global as well as developed countries could be due to the higher magnitude of injectable drug uses (IDU) in developed countries as compared with developing countries including Ethiopia which is the major causes for blood born virus (BBVs) such as HIV, hepatitis B and C viruses.

The estimated prevalence of the coinfection of HIV and HBV (0.32%), HBV and HCV (0.32%), as well as HIV and HCV (0.32%) in this study, were consistent with the reported magnitude of coinfection from a recent systematic review [45, 46]. However, the magnitude of the triple infection in this study (0.00%) was different from the reported magnitude of the global magnitude of triple infection 1.29% [45, 46]. The lower number of participants as compared with the above studies could be the possible reason for the observed variation because of the rare nature of the occurrence of triple infection requiring the large sample size to estimate the magnitude.

Regarding associated factors, consistent with previous epidemiologic studies [47], the findings of this study demonstrated that the odds of having chronic viral infections was 3.95 times greater [AOR = 3.95 (95%CI 1.18–13.17)] for those patients with severe mental illness in the age range between 30 and 40 years.

### The prevalence of undiagnosed chronic viral infections in people with severe psychiatric disorders

In this study, 20 participants had undiagnosed infected disease in patients with severe psychiatric disorders with a prevalence of 6.47% from the total participants and 76.92% of those participants with infectious disease. The rate of undiagnosed disease was relatively higher for the hepatitis B virus (80% of HBV cases) followed by hepatitis C (75% of HCV cases) and HIV (60% of HIV cases). We found that the rates of undiagnosed HCV, HBV, as well as HCV in this study, was remarkably higher as compared with the estimated undiagnosed rates of HCV, HBV, and HCV from the general population [19, 47]. Supporting this view a study conducted in Europe showed that the prevalence of undiagnosed HIV in the general population was 30% which is remarkably higher than the findings of the estimated prevalence undiagnosed HIV cases in this study (60%) [19]. Another study conducted in France revealed the prevalence of undiagnosed HIV infection in the general population to be 0.065% [48]. Similarly to HIV infection epidemiologic studies also revealed that the magnitude of 40 to 50% cases of HCV remain undiagnosed in the USA [21] whereas the reported estimate of the prevalence of undiagnosed cases of HBV infection in general population in a study conducted England was found to be 16% [22] which is remarkably lower than the results of this study where about 80% of those patients having HBV infections remained undiagnosed in people with severe mental illness.

### Suggestions for future research and clinical practice

The current study had some suggestions for future research as well as clinical practice. First, this study shows that the undiagnosed cases of chronic viral infections are remarkably higher in patients with severe psychiatric disorders. However, the data for specific categories of severe psychiatric disorders are not analyzed due to a small number of participants for the specific disorder, which suggests the need for future studies addressing these issues. Second, the prevalence of undiagnosed chronic viral infections is very high as compared with general pollution estimates which need future robust the possible reasons for the highest magnitude. Thirdly, increasing awareness using educational programs tailored to chronic viral infections among patients with severe mental illness to the patients and general public is recommended; fourthly, attention need to be given to possibly reduce the extensive level of undiagnosed viral infections among patients with severe mental illness by the concerned body with the possibilities of implementing continues medical education (CME) so that the patients will be safe from suffering related to short term and long term impacts of the undiagnosed infection-causing strong, cirrhosis and other physical and mental health-related problems; Fifthly, possible screening programs patients with severe mental illness particularly for those patients who are in high risks (young, sexually active and substance users). Furthermore, government need to have a plan aimed to early detection and interventions of HIV, HBV, and HCV infections in patients with severe mental illness; finally, studies focusing on best screening strategies to allow earlier diagnosis and treatments of HIV, HBV, and HCV infections in patients with severe mental illness who are unaware of their infection status are warranted.

### Strengths and limitations

This study had several strengths: (1) to our knowledge, this is the first study to estimate and compare the level of undiagnosed chronic viral infections including HIV, HBV, and HCV infections in patients with severe mental illness; (2) the use of standard and diagnostic instrument (SCID) to confirm the severe psychiatric disorders. Nevertheless, the current study had also some limitations: first, due to the cross-sectional nature of the study factors associated with misdiagnosis may not imply causality. Secondly, past diagnosis (previous diagnosis) was taken from the chart of the patients where there are possibilities of not recording or missing previous records which underestimate the diagnosed cases. Lastly, the small number of samples needs to be considered in interpreting the findings, particularly for the prevalence co-occurrence of chronic viral infections (the true prevalence estimates might be underestimated due to the small samples).

## Conclusion

To sum up, the current study revealed that a considerable percentage of people with severe psychiatric disorders had a diagnosis of HIV (3.24%), HBV (4.85%), HCV (1.29%), and overall viral (8.41%) infections. In this study, HBV was found to be the commonly undiagnosed viral infection (80%) followed by HCV (75%) and HIV (60%) infections. The age range between 30 and 40 years was found to be a significant predictor of chronic viral infections among patients with severe psychiatric disorders.

Early screening and treatment of HIV, HBV, as well as HCV infection, are warranted among patients with severe psychiatric disorders. Additionally, strong epidemiologic studies evaluating the reasons for the highest level of undiagnosed infectious disease among patients with SPD are recommended. Finally, Continues medical education (CME), as well as supplementary refreshment training on chronic viral infections, are suggested for the psychiatry professionals.

## Data Availability

The datasets used and analyzed during the current study are not publically available due to ethical restriction and personal data protections but are available from the corresponding author on reasonable request.

## Abbreviations

AOR: Adjusted Odds Ratio
CI: Confidence Interval
CME: Continues Medical Education
DSM: Diagnostic and Statistical Manual of Mental Disorders
ELSA: Enzyme-Linked Immunological Assay
HBsAg: Hepatitis B Suraface Antigen
HIB: Hepatitis B Virus
HIV: Human Immune Deficiency Virus
HREC: Human Research and Ethics Committee
HVC: Hepatitis C Virus
IDU: Injectable Drug Use
OR: Odds Ratio
SCID-I: Structured Clinical Interview for DSM-IV axis I Disorders
SOP: Standard Operating Procedure
SPD: Severe Psychiatric Disorders
USA: United States of America
